# Glycosyltransferase ST6GAL1 contributes to the regulation of pluripotency in human pluripotent stem cells

**DOI:** 10.1038/srep13317

**Published:** 2015-08-25

**Authors:** Yu-Chieh Wang, Jason W. Stein, Candace L. Lynch, Ha T. Tran, Chia-Yao Lee, Ronald Coleman, Adam Hatch, Victor G. Antontsev, Hun S. Chy, Carmel M. O’Brien, Shashi K. Murthy, Andrew L. Laslett, Suzanne E. Peterson, Jeanne F. Loring

**Affiliations:** 1 Department of Pharmaceutical Sciences, The University of North Texas Health Science Center, Fort Worth, Texas, USA; 2 The Scripps Research Institute, La Jolla, California, USA; 3 Center for Regenerative Medicine, The Scripps Research Institute, La Jolla, California, USA; 4 Northeastern University Boston, USA; 5 Barnett Institute of Chemical and Biological Analysis, Northeastern University, Boston, USA; 6 CSIRO, Flagship, Cell Biology Group, Clayton, Victoria, Australia; 7 Australian Regenerative Medicine Institute, Monash University, Melbourne, Victoria, Australia

## Abstract

Many studies have suggested the significance of glycosyltransferase-mediated macromolecule glycosylation in the regulation of pluripotent states in human pluripotent stem cells (hPSCs). Here, we observed that the sialyltransferase ST6GAL1 was preferentially expressed in undifferentiated hPSCs compared to non-pluripotent cells. A lectin which preferentially recognizes α-2,6 sialylated galactosides showed strong binding reactivity with undifferentiated hPSCs and their glycoproteins, and did so to a much lesser extent with differentiated cells. In addition, downregulation of ST6GAL1 in undifferentiated hPSCs led to a decrease in POU5F1 (also known as OCT4) protein and significantly altered the expression of many genes that orchestrate cell morphogenesis during differentiation. The induction of cellular pluripotency in somatic cells was substantially impeded by the shRNA-mediated suppression of ST6GAL1, partially through interference with the expression of endogenous *POU5F1* and *SOX2*. Targeting ST6GAL1 activity with a sialyltransferase inhibitor during cell reprogramming resulted in a dose-dependent reduction in the generation of human induced pluripotent stem cells (hiPSCs). Collectively, our data indicate that ST6GAL1 plays an important role in the regulation of pluripotency and differentiation in hPSCs, and the pluripotent state in human cells can be modulated using pharmacological tools to target sialyltransferase activity.

The glycosylation of macromolecules, including proteins and lipids, is critically involved in the regulation of numerous biochemical and physiological activities in eukaryotic cells^1^. It is known that glycosylation plays essential roles to ensure normal cell differentiation and embryogenesis^2^,^3^,^4^, but its function in human pluripotent stem cells (hPSCs) remains largely unclear. A recent report demonstrated that *O*-linked glycosylation influences cellular pluripotency by acting on core components of the pluripotency signaling network in mouse embryonic stem cells (mESCs)^5^, revealing a direct link between protein glycosylation and pluripotency regulation that is highly likely to exist in human cells.

Indeed, evidence suggests that protein modification by sialic acid-containing glycans and sialyltransferases may be important in the regulation of cellular pluripotency in human cells^6^,^7^,^8^,^9^. Characterization of significant differences between the glycomic composition of undifferentiated hPSCs and their differentiated derivatives revealed that sialylation is a type of glycomodification that is typically altered when hPSCs lose their pluripotency^6^,^7^. In human cells, the sialylation of macromolecules is regulated by many sialyltransferases, including ST6GAL1 (β-galactoside alpha-2,6-sialyltransferase 1)^10^,^11^. ST6GAL1 is a type II membrane protein that is typically found in the Golgi apparatus and catalyzes the transfer of sialic acid monosaccharide to galactose-containing substrates^12^. Using lectin microarrays and global gene expression profiling, we confirmed that there is a significant change in protein sialylation during differentiation in a large panel of hPSCs, and discovered the preferential expression of ST6GAL1 in undifferentiated hPSCs^8^,^13^. These results suggested that ST6GAL1 may be functionally important in hPSCs, and that changes in ST6GAL1 expression may impact the regulation of pluripotency and cell differentiation. Interestingly, another recent study has provided an additional indication that the loss of terminal sialylation on the cell surface of hPSCs may lead to neuronal differentiation^14^.

In this study, we set out to determine whether and how the sialyltransferase ST6GAL1 may be involved in the modulation of pluripotency in human cells. We used biochemical and molecular biology approaches to demonstrate that modifying ST6GAL1 expression levels in human cells induced phenotypic changes in the maintenance and establishment of pluripotency. In addition, systems biology tools were used to further dissect the signaling networks that are potentially modulated by ST6GAL1 in controlling the pluripotent state of hPSCs and cellular reprogramming.

## Materials and Methods

### Cell Culture

HDF51 (HDF-f; ScienCell Research Laboratories, Carlsbad, CA), normal mouse embryonic fibroblast (MEF), and DR4 MEF (ATCC, Manassas, VA) cells were cultured in DMEM containing 10% (15% for DR4 MEF cells) fetal bovine serum (FBS) at 37°C. HM (HEMl; ScienCell Research Laboratories, Carlsbad, CA) cells were cultured in melanocyte medium (MelM; ScienCell Research Laboratories, Carlsbad, CA). Methods for culturing undifferentiated hPSC lines used in our study were previously described^8^. WA07 and WA09 hESCs were obtained from the WiCell Stem Cell Bank (WiCell Research Institute, Madison, WI). MEL1 hESCs^15^ were provided by Dr. Andrew Laslett at CSIRO, Australia. The cells used in our study were free of mycoplasma, tested using a MycoAlert^TM^ mycoplasma detection kit (Lonza, Walkersville, MD). Additional information with regard to the cells used in our study is provided in Supplementary Table 1.

### ST6GAL1 Knockdown in hPSCs

pLKO.1 lentiviral vectors which express ST6GAL1 shRNA (shRNA2 targeting sequence: CGTGTGCTACTACTACCAG_TRCN0000035432, shRNA5 targeting sequence: CGCTGCTCTATGAGAAGAA_TRCN0000035429; Sigma Aldrich, St. Louis, MO) were used to knock down ST6GAL1 protein expression in undifferentiated hPSCs. Cells (~30% confluency) cultured in feeder-free conditions were transduced using the lentiviral vectors with and without ST6GAL1 shRNA. The transduced cells were allowed to recover in StemPro^®^ hESC serum- and feeder-free medium (Life Technologies, Carlsbad, CA) for 24 hours. StemPro^®^ hESC medium containing puromycin (2 μg/ml) was used to culture the transduced cells for an additional 3 days prior to sample collection.

### Cellular Reprogramming and Differentiation

To generate induced pluripotency in HDFs, we primarily used retroviral vectors to deliver four reprogramming factors (*POU5F1*, *SOX2*, *KLF4*, and *MYC* genes) into HDF51 cells. Methods for retrovirus-mediated cell reprogramming were previously described^8^. The transgene-free hiPSCs used in this study were generated through Sendai virus-mediated cell reprogramming. The recombinant DNA work in this study was performed according to the National Institutes of Health guidelines. To test the effect of ST6GAL1 knockdown on the establishment of pluripotency, HDFs were co-transduced with the ST6GAL1 shRNA lentiviral expression vector and the retroviral vectors for reprogramming. The transduced cells were placed onto radiation-inactivated DR4 (multiple drug resistant) MEF feeder cells at a density of 1 × 10^4^ cells per well of a six well plate and cultured for 14 days with puromycin selection (1 μg/ml for 4 days followed by 0.5 μg/ml for the rest of the culture period). To test the effect of a sialyltransferase inhibitor on the establishment of pluripotency, HDFs transduced with the retroviral vectors for reprogramming were placed onto radiation-inactivated MEF feeder cells at a density of 1 × 10^4^ cells per well of a six well plate and cultured for 14 days with 3F_ax_-peracetyl Neu5Ac, a cell-permeable sialic acid analog (Millipore, Billerica, MA). The reprogramming efficiency was evaluated using an alkaline phosphatase (AP) staining kit II (Stemgent, Cambridge, MA). To test the effect of ST6GAL1 knockdown during reprogramming, the transduced cells were placed onto Geltrex^® (^Life Technologies, Carlsbad, CA)-coated wells at a density of ~3.8 × 10^5^ (a quarter of the original cell number for transduction) cells per well of a six well plate and cultured for the indicated periods with puromycin selection. For non-directed differentiation of hPSCs by embryoid body (EB) formation, hPSCs grown on a MEF feeder layer were incubated with pre-warmed (37 °C) 300 U/ml Collagenase I (Worthington Biomedical Corp., Lakewood, NJ) in DMEM/F12 (Life Technologies, Carlsbad, CA), typically for 60–75 minutes, to yield small hPSC colony clumps in suspension and leave most of the feeder cells behind. The cell clumps were collected with minimal trituration into bFGF-deficient DMEM/F12 medium with L-glutamine containing 20% KnockOut™ Serum Replacement, 100 μM non-essential amino acids, and 100 μM ß-mercaptoethanol (hESC medium; all components from Life Technologies, Carlsbad, CA) and left to sediment by gravity for 20–30 minutes in an incubator, to enable the removal of residual MEFs from the supernatant fraction. The cells were washed, pelleted at low centrifugation speed (50 g for 2 minutes), and plated into non-adherent polystyrene petri dishes (Simport, Beloeil, Canada) in hESC medium containing 10 ng/ml bFGF and left undisturbed in an incubator for 24–48 hours to establish viable aggregate cultures before changing to differentiation culture conditions. Aggregates were collected into 25 ml conical skirt tubes (Greiner, Monroe, NC), left to sediment by gravity for ~30 minutes in an incubator, removing initial single cell debris in the supernatant, and replated to low adherence petri dishes in EB differentiation medium comprised of high glucose DMEM, 2 mM Glutamax, 1% v/v non-essential amino acids (all from Life Technologies, Carlsbad, CA) and 10% v/v fetal bovine serum (FBS), (Sigma-Aldrich, St. Louis, MO). Suspension cultures were subsequently replenished with EB differentiation medium each 3–4 days. EBs were collected into 50 ml conical tubes (BD Biosciences, San Jose, CA) following 7, 14, and 28 days of differentiation, washed twice with PBS and dissociated to single cell suspensions usingTrypLE^TM^ Express (Life Technologies, Carlsbad, CA) and a 15–30 minute incubation and gentle pipetting to assist breaking up the EB structures for the ease of flow cytometry analysis and cell sorting. The protocol used to generate melanocytic differentiated derivatives of hPSCs was reported in a previous study^16^.

### Western and Lectin-mediated Blotting

Methods for Western blotting were described in our previously published report^16^. The primary antibodies used in this study were purchased from R&D Systems (ST6GAL1; cat# AF5924), Cell Signaling (POU5F1; cat# 2840), Millipore (NANOG; cat# MABD24) and MP Biomedicals (ACTIN; cat# 08691001). HRP-conjugated secondary antibodies were from Jackson ImmunoResearch Laboratories (West Grove, PA). For SNA lectin-mediated blotting, 10 ug of total proteins from each sample were separated by SDS-PAGE and transferred onto nitrocellulose membranes. The membranes with transferred proteins were blocked using a polyvinyl alcohol solution to prevent non-specific binding. After blocking, the membranes were reacted with PBS containing Triton X-100 (0.1%) and biotinylated SNA lectin (2 μg/ml; Vector Laboratories, Burlingame, CA) at 4 °C for 2 hours. After thorough washing, the membranes were reacted with HRP-conjugated streptavidin (Jackson ImmunoResearch Laboratories, West Grove, PA) in PBS for 30 minutes. After thorough washing, the chemiluminescence of an ECL substrate catalyzed by HRP was detected using film exposure.

### Flow Cytometry and Cell Sorting

To measure SNA binding, 5 × 10^5^ cells per sample were harvested using Accutase (Life Technologies, Carlsbad, CA), pelleted, resuspended in Hank′s balanced salt solution (HBSS; Life Technologies, Carlsbad, CA) containing fluorescein-labeled SNA lectin (4 μg/ml; Vector Laboratories, Burlingame, CA), and incubated at 4°C with mild agitation for 45 minutes. At the end of the reaction, the cells were pelleted and resuspended in fresh HBSS. The fluorescence intensity of samples was analyzed using a FACSCalibur flow cytometer equipped with CellQuest Pro software (BD Biosciences, San Jose, CA). For measuring UEA-I binding and the expression of NANOG and POU5F1, aliquots of harvested cell samples were fixed using PBS containing 4% paraformaldehyde, perforated using PBS containing 0.1% Triton X-100, labeled with biotinylated UEA-I lectin (6.5 μg/ml; Vector Laboratories, Burlingame, CA) and specific antibodies targeting NANOG and POU5F1 (Millipore, Billerica, MA), and stained with fluorescein-conjugated streptavidin and secondary antibodies. The unstained, isotype and single color fluorophore controls were included for each cell sample. All tubes were kept on ice during lectin and antibody labeling procedures, and prior to multiple color analyses on a LSRII flow cytometry analyzer (BD Biosciences, San Jose, CA). A routine hPSC maintenance culture was harvested with TrypLE^TM^ Express to provide a Day 0 control for each analysis. For the subsequent collection of UEA-I positive and negative live cell populations from Day 0, 7,14 and 28 cultures of EB differentiation, cell suspensions were labeled only with the biotinylated UEA-I lectin and detected with fluorescein-conjugated streptavidin. Two populations of cells in each sample were gated and sorted on a FACSVantage Diva cell sorter (BD Biosciences, San Jose, CA). UEA-I positive and negative cell fractions of 100,000–700,000 (relative to differentiation state) were collected and pelleted for global gene expression profiling. For detecting apoptosis, 5 × 10^5^ cells from each sample were stained using a dead cell apoptosis Kit (Alexa Fluor 488-conjugated annexin V and propidium iodide; Life Technologies, Carlsbad, CA) according to the manufacturer’s instruction. The fluorescence intensity indicating apoptotic cell death was analyzed using a LSRII flow cytometry analyzer (BD Biosciences, San Jose, CA).

### Global Gene Expression and qRT-PCR Analysis

Global gene expression profiling was performed using HT-12v4 Human Gene Expression BeadChips (Illumina, Hayward, CA), according to the manufacturer’s instructions. Data were filtered for detection *P* value <0.01 in GenomeStudio (Illumina, Hayward, CA), and normalized using the LUMI package with RSN (Robust spline normalization) algorithm in R. Qlucore Omics Explorer was used to perform differential gene expression analysis and hierarchical clustering. The ontology analysis of differentially expressed genes was performed using Genomic Regions Enrichment of Annotations Tool (GREAT, http://bejerano.stanford.edu/great/public/html/). To validate the expression of genes that were differentially expressed in distinct samples, cDNA of each RNA sample was first synthesized using the QuantiTect Reverse Transcription Kit (Qiagen, Valencia, CA). Multiplex qRT-PCR was performed using Taqman^®^ assays for *ST6GAL1*, *PITX2* and *KAT6A* genes (cat# Hs00949382_m1, Hs04234069_mH and Hs01063029_m1; Life Technologies, Carlsbad, CA), according to the manufacturer’s instructions. To measure the expression of endogenous *POU5F1*, *SOX2*, *KLF4* and *MYC* genes, qRT-PCR was performed after cDNA synthesis using SYBR^®^ Green PCR Master Mix (Life Technologies, Carlsbad, CA) and specific primers that target untranslated regions of four endogenous gene transcripts (absent in exogenously expressed reprogramming factors). The specific primers that target endogenous transcripts included: KLF4 3′UTR_For (^5′^TATGACCCACACTGCCAGAA^3′^), KLF4 ^3′^UTR_Rev (^5′^ATCCAGTCACAGACCCCATC^3′^), SOX2 endo_S1430 (^5′^GGGAAATGGGAGGGGTGCAAA^3′^), SOX2 endo_AS1555 (^5′^TTGCGTGAGTGTGGATGGGAT^3′^), MYC 3^′^UTR_For (^5′^CGGAACTCTTGTGCGTAAGG^3′^), MYC 3′UTR_Rev (^5′^CTCAGCCAAGGTTGTGAGGT^3′^), POU5F1 ^3′^UTR_For (^5′^GTACTCCTCGGTCCCTTTCC^3′^), POU5F1 ^3′^UTR_Rev (^5′^CAAAAACCCTGGCACAACT^3′^).

### Analysis of SNA lectin binding affinity and kinetics

Methods for functionalizing microfluidic channels with lectins and using a microfluidic device to examine the affinity and kinetics of specific lectin binding to cells were described in a previously published report^17^.

## Results

### ST6GAL1 is highly expressed and active in undifferentiated hPSCs

**Figure 1:**
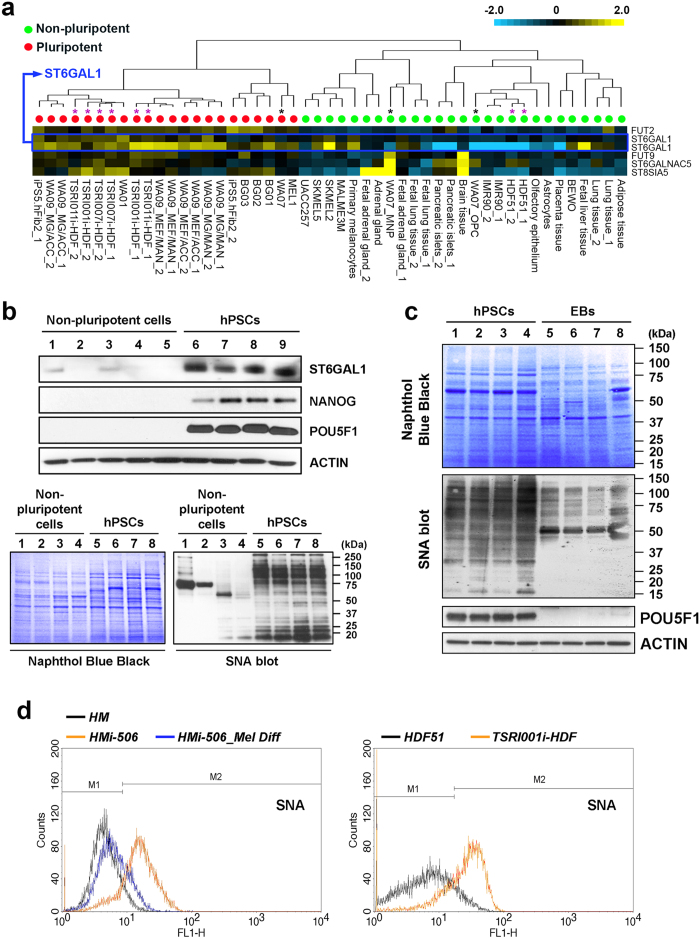
ST6GAL1 is preferentially expressed and active in hPSCs. (**a**) Heatmap representation of ST6GAL1 transcript expression, as well as other glycosyltransferases, measured by global gene expression analysis in hPSCs and non-pluripotent cell samples. The expression of ST6GAL1 in each cell sample, detected by two independent probes targeting different regions of the ST6GAL1 transcript is shown. *Black asterisk:* WA07 hESCs and their isogenic differentiated derivatives. *Purple asterisk:* HDF51 cells and hiPSCs generated from HDF51 cells. (**b**) *Upper panel*: Western blotting analysis of ST6GAL1 in cell samples showed that undifferentiated hPSCs had higher ST6GAL1 protein expression, compared to differentiated cells. *Lane 1:* HMi-506_Mel Diff^16^, *2:* HDF68i-505_Mel Diff^16^, *3:* HM (HEMl), *4:* HDF68, *5:* HDF51, *6:* WA09, *7:* HDF68i-505^16^, *8:* HMi-506^16^, *9:* TSRI001i-HDF^8^. *Lower panel:* SNA-mediated blotting showed that protein samples extracted from undifferentiated hPSCs had higher reactivity to SNA binding. *Lane 1:* HM, *2:* HDF68i-505_Mel Diff, *3:* HDF51, *4:* HDF68, *5:* WA09, *6:* HMi-506, *7:* TSRI001i-HDF, *8:* HDF68i-505. (**c**) SNA-mediated blotting showed that protein samples extracted from derivatives of undirected differentiation (embryoid bodies, EBs) had less reactivity to SNA, compared to those from paired, undifferentiated hPSCs. *Lane 1:* WA09, *2:* WA07, *3:* TSRI001i-HDF, *4:* HMi-506, *5:* WA09_EBs, *6:* WA07_EBs, *7:* TSRI001i-HDF_EBs, *8:* HMi-506_EBs. (**d**) Flow cytometric analysis demonstrated that live human cells in the pluripotent state were more reactive to SNA at the cellular level.

To begin to address the significance of ST6GAL1 expression in the regulation of pluripotent states in human cells, we analyzed ST6GAL1 mRNA and protein expression in multiple undifferentiated hPSC lines [including human embryonic stem cells (hESCs) and induced pluripotent stem cells (hiPSCs)], their differentiated derivatives, and somatic cells that were used for generating the hiPSCs. In particular, we examined several hESC and hiPSC lines paired with their differentiated derivatives. Analysis of gene expression using both microarrays and quantitative RT-PCR (qRT-PCR) showed that cells in the pluripotent state generally had higher expression of the ST6GAL1 transcript (Fig. 1a and Supplementary Figure 1), despite a noticeable variation of ST6GAL1 mRNA expression among different hPSC lines. This higher expression of ST6GAL1 in undifferentiated hPSCs was also reflected at the protein level (Fig. 1b upper panel), consistent with a previous finding^18^. Since ST6GAL1 catalyzes the terminal addition of sialic acid to β-galactosides to form α-2,6 sialylated glycoconjugates that can be selectively bound by the SNA lectin^19^, we used SNA as a probe to detect enzymatic products of ST6GAL1 in protein samples isolated from hPSCs and non-pluripotent cells. The SNA-mediated blotting revealed that proteins in undifferentiated hPSCs had distinguishably higher reactivity to SNA, compared to those in non-pluripotent cells (Fig. 1b lower panel and 1c). At the cellular level, hPSCs also showed higher reactivity to SNA binding, compared to their non-pluripotent counterparts (Fig. 1d and Supplementary Figure 2). These results indicate that not only is ST6GAL1 highly expressed but it is also enzymatically active in undifferentiated hPSCs.

### Effects of loss of ST6GAL1 function in undifferentiated hPSCs

**Figure 2:**
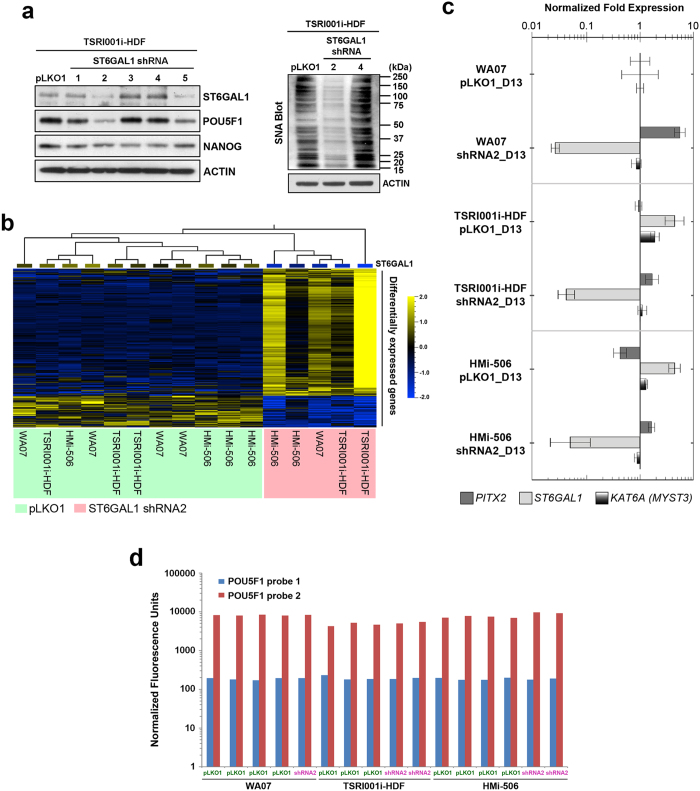
Downregulation of ST6GAL1 in hPSCs has an impact on signaling networks involved in pluripotency regulation and embryogenesis. (**a**) *Left Panel:* Western blotting analysis showed that two independent shRNA sequences (shRNA2 and shRNA5) that target ST6GAL1 transcripts led to effective downregulation of ST6GAL1 protein 72 hours after transduction. While the protein level of NANOG was relatively unaffected, the protein level of POU5F1 was decreased in hPSCs that received shRNA2 and shRNA5. *Right Panel:* SNA-mediated blotting showed that protein samples extracted from hPSCs which received shRNA2 had lower reactivity to SNA, indicating a decreased amount of α-2,6 sialylated glycoconjugates in the cells. *pLKO1:* the empty factor control for the transduction of shRNA expression vectors. (**b**) Global gene expression profiling followed by differential gene expression analysis revealed a group of genes (~400 genes) that were differentially expressed (*P* < 0.01, *F*-test) in multiple lines of hPSCs with and without ST6GAL1 knockdown. (**c**) Quantitative RT-PCR was used to validate the expression of a selection of differentially expressed genes, confirming the upregulation of the *PITX2* gene and the downregulation of *KATA6A* and *ST6GAL1* genes due to ST6GAL1 knockdown. *D13:* 13 days after the beginning of puromycin selection (14 days post transduction). (**d**) The expression level of POU5F1 transcripts reflected by normalized fluorescence units in expression array analysis indicated that the *POU5F1* gene expression was relatively unchanged at the transcriptional level in multiple lines of hPSCs with ST6GAL1 knockdown.

We identified two independent shRNA sequences (shRNA2 and shRNA5) that reduced ST6GAL1 protein expression in hPSCs by more than 60% (Fig. 2a). Despite the fact that NANOG protein levels were relatively unaffected by ST6GAL1 knockdown in hiPSCs after 72 hours, the reduction of ST6GAL1 induced by both shRNA sequences was paralleled by decreases in POU5F1 (a.k.a. OCT4) protein and SNA reactivity (Fig. 2a) in the cells. This suggests that the pluripotency signaling network in hPSCs can be partially modulated by ST6GAL1. Using global gene expression profiling followed by differential expression analysis, we further identified a group of genes (~400 genes) that were significantly upregulated or downregulated in hPSCs in which *ST6GAL1* had been knocked down (Fig. 2b,c and Supplementary Figure 3a). Notably, many genes (*e.g*., *COL6A3*, *S100A4*, *SNAI2*, *HOXC8*, *PITX2*, and *TWIST2*^20^,^21^,^22^,^23^,^24^,^25^) known to be involved in organ development or cell morphogenesis during differentiation were upregulated by ST6GAL1 knockdown (Table 1), while the transcripts of genes (*e.g*., *KAT6A* (MYST3^26^)) known to be associated with pluripotency were downregulated. Genes including *PTPRM and GNG11* that appeared as potential regulators of cellular pluripotency in a genome-wide RNAi screening^27^ were differentially expressed in hPSCs with ST6GAL1 knockdown (Supplementary Table 2). The ontology analysis of the differentially expressed genes showed that they are highly enriched in the regulation of biological processes relevant to cell differentiation and organogenesis (Supplementary Figure 4). This suggests that ST6GAL1 knockdown may bias cellular pluripotency and differentiation capacity in hPSCs through its global impact on the expression of genes involved in multiple cell lineages. Although POU5F1 protein was reduced in hPSCs with successful knockdown of ST6GAL1, we did not find similarly reduced expression of the *POU5F1* gene at the transcriptional level (Fig. 2d), suggesting that the ST6GAL1 knockdown-induced alteration of POU5F1 protein may work through a post-transcriptional or post-translational mechanism.

### Effects of loss of ST6GAL1 function during cellular reprogramming

**Figure 3:**
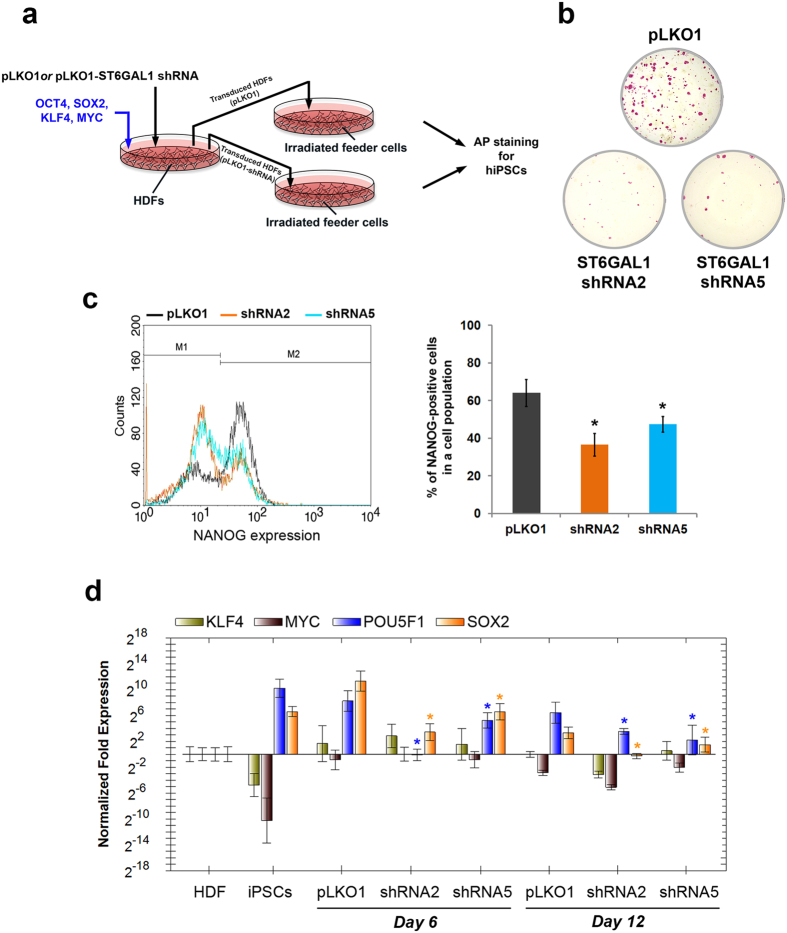
ST6GAL1 knockdown impedes cellular reprogramming and establishment of induced pluripotency in human somatic cells. (**a**) Schematic illustration of the experimental strategy to examine the influence of ST6GAL1 knockdown on cellular reprogramming. Twenty-four hours after HDFs received POU5F1, SOX2, KLF4, and MYC with or without ST6GAL1 shRNA, the transduced HDFs were seeded on X-ray irradiated feeder cells (mouse embryonic fibroblasts, MEFs). Fourteen days later, alkaline phosphatase (AP) staining was used to examine hiPSC colonies formed by transduced HDFs on the feeder cells. (**b**) AP staining showed that dramatically fewer hiPSC colonies with AP activity were obtained from cellular reprogramming under ST6GAL1 knockdown mediated by shRNA2 and shRNA5. (**c**) Quantitative analysis of NANOG expressing cells in the reprogrammed cell population showed that shRNA2 and shRNA5 both led to a significant reduction in NANOG expressing cells in the analyzed cell populations. *Left Panel:* The histogram representation of flow cytometry analysis. *Right panel:* the quantitative result of flow cytometry analysis (*n* = 3; *P < 0.05, *t*-test). (**d**) The expression of endogenous *POU5F1*, *SOX2*, *KLF4* and *MYC* genes in cells that underwent reprogramming with or without ST6GAL1 knockdown was measured by qRT-PCR with primer sets that target untranslated regions of the endogenous gene transcripts. The induction of endogenous *POU5F1* and *SOX2* gene expression at the early stage of cellular reprogramming in HDFs was substantially suppressed by shRNA-mediated ST6GAL1 knockdown (*n* = 3; *P < 0.05, *t*-test). *Day 6:* cell samples collected at 6 days after the initial transduction (2 days after the beginning of puromycin selection). *Day 12:* cell samples collected at 12 days after the initial transduction (8 days after the beginning of puromycin selection).

Having demonstrated that ST6GAL1 is preferentially expressed in *bona fide* pluripotent cells and functionally involved in the regulation of their pluripotency signaling, we wanted to understand whether ST6GAL1 also plays a role in the establishment of induced pluripotency in somatic cells. We performed cell reprogramming in human dermal fibroblasts (HDFs) using four transcription factors (*POU5F1*, *SOX2*, *KLF4*, and *MYC*) with and without shRNA-mediated ST6GAL1 knockdown (Fig. 3a). Reprogramming efficiency was measured by alkaline phosphatase staining to detect hiPSC colonies and flow cytometric analysis of NANOG expression. As shown in Fig. 3b, the shRNA targeting ST6GAL1 dramatically diminished the number of hiPSC colonies induced by the reprogramming factors. Also, the induction of endogenous NANOG expression in the reprogrammed cells was significantly reduced by the shRNA (Fig. 3c). These results suggest that ST6GAL1 is crucial for the establishment of pluripotency, and that induced pluripotency may be impaired if ST6GAL1 activity is not present during reprogramming. Consistent with the reduced induction of NANOG expression, the expression of endogenous *POU5F1* and *SOX2* at the initial stage of reprogramming was greatly suppressed by ST6GAL1 knockdown (Fig. 3d), providing a possible mechanistic explanation for the inefficiency of generating hiPSCs in the absence of ST6GAL1. Interestingly, POU5F1 and SOX2 have been shown to counteract lineage specification signaling and interact with each other and other factors to orchestrate self-renewal and pluripotency in hPSCs^28^,^29^,^30^. Our results therefore suggest that ST6GAL1 may regulate the establishment of induced pluripotency by optimizing the balance of POU5F1 and SOX2 during the reprogramming process.

### ST6GAL1 knockdown impedes cellular reprogramming through multiple mechanisms

To further dissect the signaling networks modulated by ST6GAL1 in cells undergoing reprogramming, we analyzed the global gene expression profiles of HDFs that were reprogrammed while ST6GAL1 was knocked down. Differential expression analysis revealed that a group of genes (~570 genes) were differentially expressed in the reprogrammed cells with and without ST6GAL1 knockdown (Supplementary Figure 5a and 5b). Many of these genes are involved in ribosomal biogenesis and the post-transcriptional regulation of gene expression (*e.g*., RNA processing and translation; Supplementary Figure 5c), indicated by gene ontology analysis. It has been reported that alterations in ribosome synthesis (ribosomal stress) and decreases in ribosome levels may cause cell cycle arrest through both TP53-dependent and TP53-independent mechanisms^31^,^32^. In addition, cell cycle arrest is a well-documented barrier to the induction of cellular pluripotency^33^,^34^,^35^,^36^. Thus, our findings suggest that ribosomal stress-mediated inhibition of cell proliferation may also contribute to the decreased reprogramming efficiency caused by ST6GAL1 knockdown.

### A sialyltransferase inhibitor reduces the efficiency of cellular reprogramming

**Figure 4:**
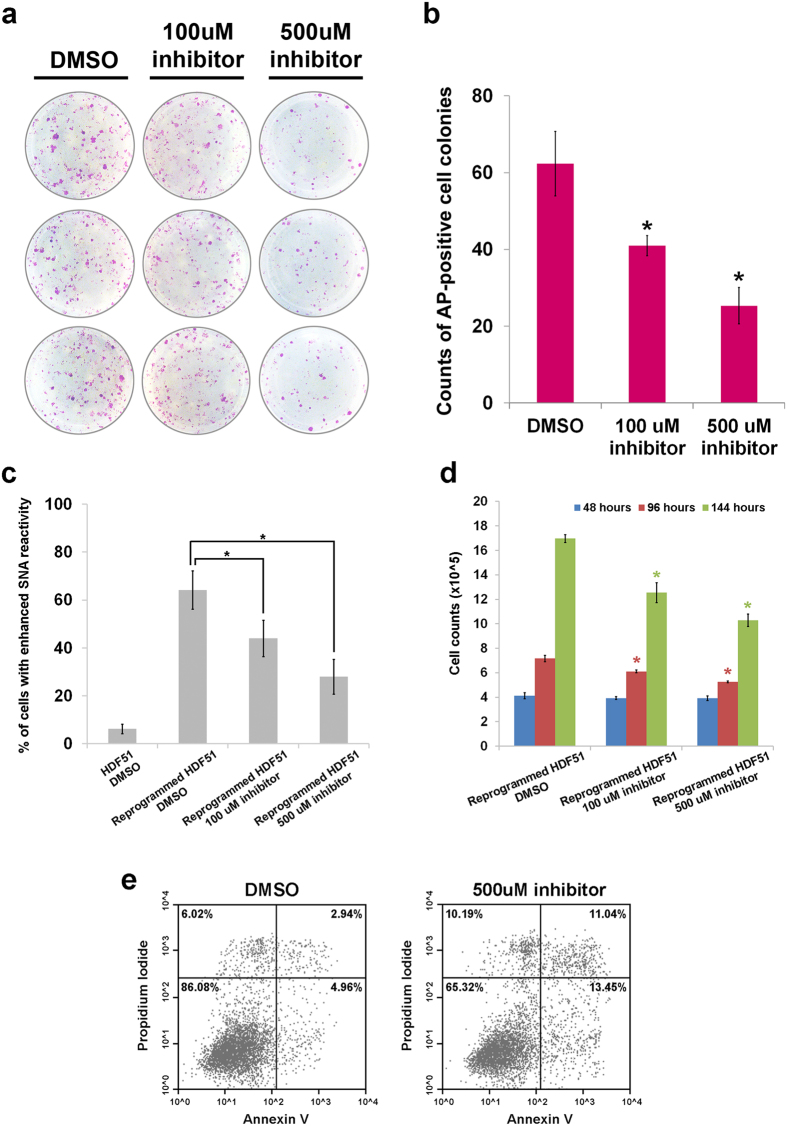
Treatment of a sialyltransferase inhibitor suppresses cellular reprogramming and establishment of induced pluripotency in human somatic cells. (**a**) AP staining showed that fewer hiPSC colonies with AP activity were obtained from cellular reprogramming under the treatment of 3F_ax_-peracetyl Neu5Ac. (**b**) The numbers of AP-positive colonies with a size equal or larger than 1.0 mm in diameter were counted at the end of reprogramming experiments, which showed a dose-dependent reduction of hiPSC formation due to the treatment of 3F_ax_-peracetyl Neu5Ac (*n* = 3; *P < 0.05, *t*-test). (**c**) The percentages of cells with enhanced SNA reactivity in reprogrammed HDF51 cells were significantly reduced by the treatment of 3F_ax_-peracetyl Neu5Ac for 6 days in a dose-dependent manner (*n* = 3; *P < 0.05, *t*-test), indicating decreased sialylation on the cell surface. (**d**) The cell counts of reprogrammed HDF51 cells at different time points over 6 days were marginally but significantly reduced by the treatment of 3F_ax_-peracetyl Neu5Ac in a dose-dependent manner (*n* = 3; *P < 0.05, *t*-test), indicating a slight suppression of cell proliferation. (**e**) The number of apoptotic cells in reprogrammed HDF51 cells was slightly increased by the treatment of 500 μM 3F_ax_-peracetyl Neu5Ac for 6 days.

In light of the reduction in reprogramming efficiency due to ST6GAL1 knockdown, we used a cell-permeable sialyltransferase inhibitor, 3F_ax_-peracetyl Neu5Ac, to test whether it would also suppress the establishment of cellular pluripotency in somatic cells. Rillahan *et al*. have reported significant inhibition of ST6GAL1-mediated sialylation in HL-60 cells treated with 200 uM 3F_ax_-peracetyl Neu5Ac^37^. As shown in Fig. 4a,b, treatment with the sialyltransferase inhibitor during cell reprogramming reduced the number of AP-positive cell colonies in a dose-dependent manner. The inhibitor-induced reduction in reprogramming efficiency was concordant with the decreased SNA reactivity (Fig. 4c). In addition, treatment with the inhibitor led to a slight but significant reduction in proliferative capacity and viability of the cells (Figs 4d,e). Although the shRNA-mediated ST6GAL1 knockdown repressed induction of pluripotency to a greater extent than the drug treatment (Fig. 3b and Supplementary Figure 3b), our results suggest that the modulation of cellular pluripotency can be achieved using pharmacological tools to target sialyltransferase activity.

## Discussion

The pivotal roles of sialic acid and sialyltransferase-mediated macromolecule sialylation in many physiological and pathological processes, including the development of embryos, cancer progression, the regulation of immune systems, and host-pathogen interactions, have been identified by numerous studies^38^,^39^,^40^. In our study, we demonstrated that the downregulation of ST6GAL1 in undifferentiated hPSCs biases the core signaling of cellular pluripotency and significantly alters the expression of many genes that orchestrate cellular differentiation and organ development. Most interestingly, the induction of cellular pluripotency in somatic cells was dramatically impeded by ST6GAL1 suppression, likely through the perturbation of endogenous POU5F1 and SOX2 expression as well as inhibition of cell proliferation.

To assess variation among different hPSC lines^41^,^42^,^43^,^44^, the expression of ST6GAL1 was analyzed in a broad spectrum of pluripotent and non-pluripotent cell samples including a variety of hPSC lines paired with isogenic differentiated derivatives and the primary cells used for reprogramming. Despite variation in levels of ST6GAL1 mRNA expression among different hPSC lines, the undifferentiated pluripotent cells consistently showed higher ST6GAL1 expression compared with the differentiated derivatives and primary cells. In addition, protein samples extracted from differentiated cells have relatively low binding reactivity with the SNA lectin, compared with those from isogenic undifferentiated hPSCs. Our results suggest that high levels of ST6GAL1 expression and enzymatic activity are specifically associated with pluripotency in human cells.

We sought to determine what mechanisms may underlie the effect of ST6GAL1 on pluripotency. Using global gene expression profiling followed by differential gene expression analysis, we identified a group of genes that are upregulated in response to ST6GAL1 knockdown in undifferentiated hPSCs. The gene ontology analysis indicated that many of these genes are involved in the regulation of developmental processes and appear to influence multiple cell lineages. Although additional study is needed to determine whether these changes in gene expression are primarily mediated through biasing the pluripotency core signaling, our data suggest a role of ST6GAL1 in the negative regulation of differentiation signaling that maintains pluripotency in hPSCs. Published work in other systems has shown that the α-2,6 sialylation mediated by ST6GAL1 is associated with a less differentiated phenotype in mouse mammary tumors^12^ and enhanced PI3K/AKT signaling in human cancer cells^45^,^46^. Since PI3K/AKT activity plays an important role in the maintenance of self-renewal in hPSCs by restraining prodifferentiation signaling^47^, it appears possible that the suppression of ST6GAL1 activity may promote differentiation by interfering with PI3K/AKT signaling in hPSCs.

Our findings regarding the ST6GAL1 knockdown-mediated downregulation of POU5F1 protein that occurred without an obvious decrease of POU5F1 mRNA indicate that a post-transcriptional or post-translational mechanism controlling POU5F1 expression may be regulated through ST6GAL1. The expression of genes involved in protein synthesis or quality control mechanisms in undifferentiated hPSCs did not seem particularly affected by ST6GAL1 knockdown. However, we have observed the expression of genes highly relevant to ribosomal biogenesis and the post-transcriptional regulation of gene expression are particularly disturbed by ST6GAL1 knockdown in somatic cells undergoing cell reprogramming. Catalyzed by another glycosyltransferase (*O*-GlcNAc transferase), the post-translational *O*-GlcNAcylation of POU5F1 significantly influences the activity of POU5F1 in mESCs^5^. Although additional investigation would be needed to elucidate mechanistic details, our data together with other studies indeed support the likelihood of post-transcriptional or post-translational mechanisms mediated by the sialyltransferase ST6GAL1 in controlling the protein level and function of POU5F1 in hPSCs.

ST6GAL1 knockdown affected cellular reprogramming, reducing the number of reprogrammed cells. This was accompanied by the altered expression of genes involved in ribosomal biogenesis and RNA processing in addition to the repression of endogenous POU5F1 and SOX2 expression in the reprogrammed somatic cells. Consistent with our results, several recent reports have described the finding that interference with ribosome homeostasis (ribosomal stress) led to growth delay, apoptosis and differentiation in murine pluripotent stem cells^48^,^49^. Thus, our results suggest that ST6GAL1 activity may help to stabilize pluripotency by preventing ribosomal stress during the reprogramming process in hPSCs.

To determine the effects of blocking the enzymatic activity of ST6GAL1 without altering its expression, we used a newly-developed sialic acid analog (3F_ax_-peracetyl Neu5Ac) that has been extensively characterized in biochemical and cell-based analyses^37^. The inhibitor did reduce reprogramming efficiency, but not as much as the effect observed by knocking down ST6GAL1 expression. This may be due to the incomplete suppression of ST6GAL1 activity and the inactivation of another enzyme, ST3GAL1, which is known to occur in live cells treated with 3F_ax_-peracetyl Neu5Ac^37^. Our previous study revealed that ST3GAL1 is preferentially expressed in many differentiated cells, including the HDFs that we used for the reprogramming studies^8^. Thus, it seems possible that suppression of ST3GAL1 may counteract the effects of the loss of ST6GAL1, allowing more cells to be reprogrammed. A more specific and potent small-molecule inhibitor may better mimic the effect of shRNA targeting ST6GAL1 in the regulation of cellular pluripotency.

## Conclusions

In this study, we have shown that ST6GAL1 is critically involved in the regulation of cellular pluripotency and essential for efficient induction of pluripotency in somatic cells, potentially through multiple mechanisms. To our knowledge, this work provides the first direct evidence for the functional significance of a sialyltransferase and macromolecule sialylation in regulating the pluripotent state in human cells. Targeting glycosyltransferases and protein glycosylation could be a useful strategy for manipulating hPSCs for research and regenerative medicine.

## Additional Information

**How to cite this article**: Wang, Y.-C. *et al*. Glycosyltransferase ST6GAL1 contributes to the regulation of pluripotency in human pluripotent stem cells. *Sci. Rep*. **5**, 13317; doi: 10.1038/srep13317 (2015).

## Supplementary Material

Supplementary Information

## Figures and Tables

**Table 1:** Genes that are involved in cell or organ morphogenesis and differentially expressed in hPSCs with ST6GAL1 knockdown.

Gene Name	Fold Change (knockdown/control)	*P* value (*F*-test)
ANTXR1	1.358615	0.004427
COL3A1	18.10201	3.27E-07
COL6A2	1.877785	0.002152
COL6A3	4.273398	0.00015
DAB2	4.075949	0.000344
DKK1	1.911963	0.004
FN1	2.018123	0.001211
MEG3	2.525871	0.000155
NTF3	1.498846	0.003855
PRSS23	3.534211	0.001322
RPS6KA2	1.768516	0.00018
S100A4	6.758956	1.93E-05
S100A6	3.890132	0.000399
SLIT3	2.238777	0.001546
SNAI2	4.19717	6.73E-05
TRAK1	1.606	0.001001
TSPO	1.745324	0.004938
ANGPT1	1.608027	0.000904
ARID5B	3.490712	0.000275
CD44	3.305001	0.004976
COL1A2	5.251568	3.90E-06
COL5A1	5.055658	0.000118
COL8A1	6.030956	0.001054
COMP	2.422019	0.000522
CRYGB	1.326064	8.74E-05
DCN	58.4075	6.95E-10
ENG	1.674513	0.001303
FZD1	1.20207	0.001537
GAS1	5.137223	0.000236
GREM1	9.592929	9.73E-05
HEG1	2.404444	0.000406
HOXA5	1.164782	0.003494
HOXC4	1.773052	0.001589
HOXC8	4.19109	0.000182
ID2	2.850354	0.003447
IGFBP5	2.114798	5.94E-05
MEF2D	0.813538	0.003326
MSX1	3.0921	0.004755
MYLK	1.946184	0.004114
NNMT	7.347762	4.22E-05
NRP1	2.751723	0.000539
OSR1	2.245728	0.001449
PDGFRA	2.562541	0.000506
PHACTR3	2.016834	0.002722
PITX2	1.47441	0.000332
PTPRM	1.444159	0.004339
RDH10	1.565426	0.002461
RTN4	0.857992	0.00114
TGFBR2	1.583883	0.002067
TSHZ1	1.545548	0.003036
TWIST2	2.225789	0.001767
TXNDC5	1.195257	0.003299
VEGFC	1.696232	0.001064
