# Supplementary material for: Glycosyltransferase ST6GAL1 contributes to the regulation of pluripotency in human pluripotent stem cells
Source: Sci Rep. 2015 Aug 25;5:13317. doi: 10.1038/srep13317 (PMC4548446; doi:10.1038/srep13317)
Supplement: Supplementary Information [file srep13317-s1.pdf]

Running head: ST6GAL1 regulates cellular pluripotency

Glycosyltransferase ST6GAL1 contributes to the regulation of pluripotency in human pluripotent stem cells

Yu-Chieh Wang*¶[1,2,3], Jason W. Stein*[2,3], Candace L. Lynch[2,3], Ha T. Tran[2,3], Chia-Yao Lee[2,3], Ronald Coleman[2,3], Adam Hatch[4], Victor G. Antontsev[4], Hun S. Chy[6,7], Carmel O'Brien[6,7], Shashi K. Murthy[4,5], Andrew L. Laslett[6,7], Suzanne E. Peterson[2,3], Jeanne F. Loring[2,3]

[1] Department of Pharmaceutical Sciences, The University of North Texas Health Science Center, Fort Worth, Texas, USA

[2] Department of Chemical Physiology, [3] Center for Regenerative Medicine, The Scripps Research Institute, La Jolla, California, USA

[4] Department of Chemical Engineering, [5] Barnett Institute of Chemical and Biological Analysis, Northeastern University, Boston, USA

[6] CSIRO, Manufacturing Flagship, Cell Biology Group, Clayton, Victoria, Australia

[7] Australian Regenerative Medicine Institute, Monash University, Melbourne, Victoria, Australia

* These authors contribute equally to this work

¶ Correspondence should be addressed to Y.-C. Wang

Yu-Chieh Wang, Ph.D.
Department of Pharmaceutical Sciences
The University of North Texas Health Science Center
3500 Camp Bowie Boulevard, RES-314G
Fort Worth, Texas 76107
Tel: (817) 735 2944
Fax: (817) 735 2603
Email: yu-chieh.wang@unthsc.edu

Supplementary Figure Legends

Supplementary Figure 1. Expression of the *ST6GAL1* gene transcript was generally lower in non-pluripotent cells, tissues and differentiated derivatives of hPSCs. (**a**) Expression of the *ST6GAL1* gene transcript was measured by qRT-PCR in a panel of pluripotent and non-pluripotent samples. Notably, this particular panel of samples consisted of multiple pairs of pluripotent and differentiated samples (*e.g.,* WA09 with WA09_Hepato Diff, WA07 with WA07_Cardimyo Diff, WA07 with WA07_MNP, WA01 with WA01_NPC Diff, SuBiHD1 with SuBiHD1_Striatal Diff, SuBi1 with SuBi1_Striatal Diff, HMi-506 with HM, and HDF51i-509 with HDF51) that are isogenic but differ in regard to pluripotency. (**b**) EBs made with MEL1 and WA09 hESCs were analyzed by flow cytometry using a pluripotency-associated lectin, UEA-I[8], and a specific antibody against POU5F1 to confirm pluripotency loss in cells within the EBs. The cells in late-stages of differentiation (Day 14 and 28) showed decreased reactivity with UEA-I and almost complete depletion of POU5F1 expression. Two subpopulations of cells (UEA-I-positive cells and UEA-I-negative cells) were sorted and collected from the same batch of EBs on day 0, 14 and 28 of EB differentiation. (**c**) Heatmap representation of expression of selected pluripotency-relevant genes and *ST6GAL1* measured in gene expression array analysis revealed that UEAI-negative cells and UEAI-positive cells in the late-stage EBs of MEL1 and WA09 hESCs were broadly losing pluripotency signaling and *ST6GAL1* expression.

Supplementary Figure 2. The binding affinity and kinetics between SNA lectin and cell samples were analyzed using lectin-functionalized microfluidic channels. (**a**) Schematic illustration of flow chamber geometry and shear stress profile for the microfluidic device used in our study. Cell samples were steadily flowed from inlets into channels that were pre-functionalized with lectins AAL, AOL, LTL, SNA, SSA, and TJA-II. As cells flowed through the channel, they were subjected to a linear gradient in fluid shear stress and allowed to interact with surface-immobilized lectins as a function of decreasing fluid shear stress. (**b**) HMi-506

hiPSCs were reprogrammed from HM cells and differentiated into melanocytic derivatives (HMi-506_Mel Diff cells). The binding affinity analysis indicated that undifferentiated HMi-506 hiPSCs had significantly higher binding affinity with SNA lectin (n=3; *P<0.05, t-test), compared to non-pluripotent HM and HMi-506_Mel Diff cells. The level of binding affinity was reflected by the number of cells attached to different areas in the channels.

Supplementary Figure 3. Gene expression analysis in hiPSCs with the expression of negative control shRNA and reprogrammed cells with the treatment of sialyltransferase inhibitor $3F_{ax}$-peracetyl Neu5Ac. (**a**) Quantitative RT-PCR was used to measure the expression of selected genes, confirming the upregulation of *PITX2* gene and the downregulation of *KATA6A* and *ST6GAL1* genes specifically due to the effective shRNA sequence (shRNA2) targeting ST6GAL1. *D12:* 12 days after the beginning of puromycin selection (13 days post transduction). (**b**) The expression of endogenous *POU5F1*, *SOX2*, *KLF4* and *MYC* genes in HDF51 cells that underwent reprogramming with ST6GAL1 knockdown (shRNA2) and the treatment of 500µM $3F_{ax}$-peracetyl Neu5Ac ($3F_{ax}$ Neu5Ac) was measured by qRT-PCR with primer sets that target untranslated regions of the endogenous gene transcripts. *Day 6:* cell samples collected at 6 days after the initial transduction (2 days after the beginning of puromycin selection for cells receiving pLKO1 shRNA expression vectors or 2 days after the beginning of treatment for cells receiving DMSO and inhibitor).

Supplementary Figure 4. Gene ontology (GO) analysis revealed that the genes differentially regulated by ST6GAL1 knockdown were highly enriched in biological processes including critical developmental events (highlighted by red asterisks).

Supplementary Figure 5. ST6GAL1 knockdown leads to significant alterations of gene expression involved in ribosomal biogenesis and the post-transcriptional regulation in HDFs that undergo cellular reprogramming to acquire induced pluripotency. (**a**) Global gene expression profiling followed by differential gene expression analysis revealed a group of genes (~570

genes) that were differentially expressed (*P*<0.001, *F*-test) in HDFs reprogrammed using four transcription factors with and without shRNA-mediated ST6GAL1 knockdown. *D6:* cell samples collected at 6 days after the initial transduction (2 days after the beginning of puromycin selection). *D12:* cell samples collected at 12 days after the initial transduction (8 days after the beginning of puromycin selection). *D16:* cell samples collected at 16 days after the initial transduction (12 days after the beginning of puromycin selection). (**b**) Principal component analysis (PCA) based on the differentially expressed genes in the cell samples that received control (pLKO1) and ST6GAL1 shRNA (shRNA2 and shRNA5) vectors. (**c**) Gene ontology (GO) analysis revealed that the genes that were differentially expressed due to ST6GAL1 knockdown were highly involved in biological processes including ribosomal biogenesis, RNA processing and translation (highlighted by red asterisks).

Supplementary Figure 1_Wang et. al.

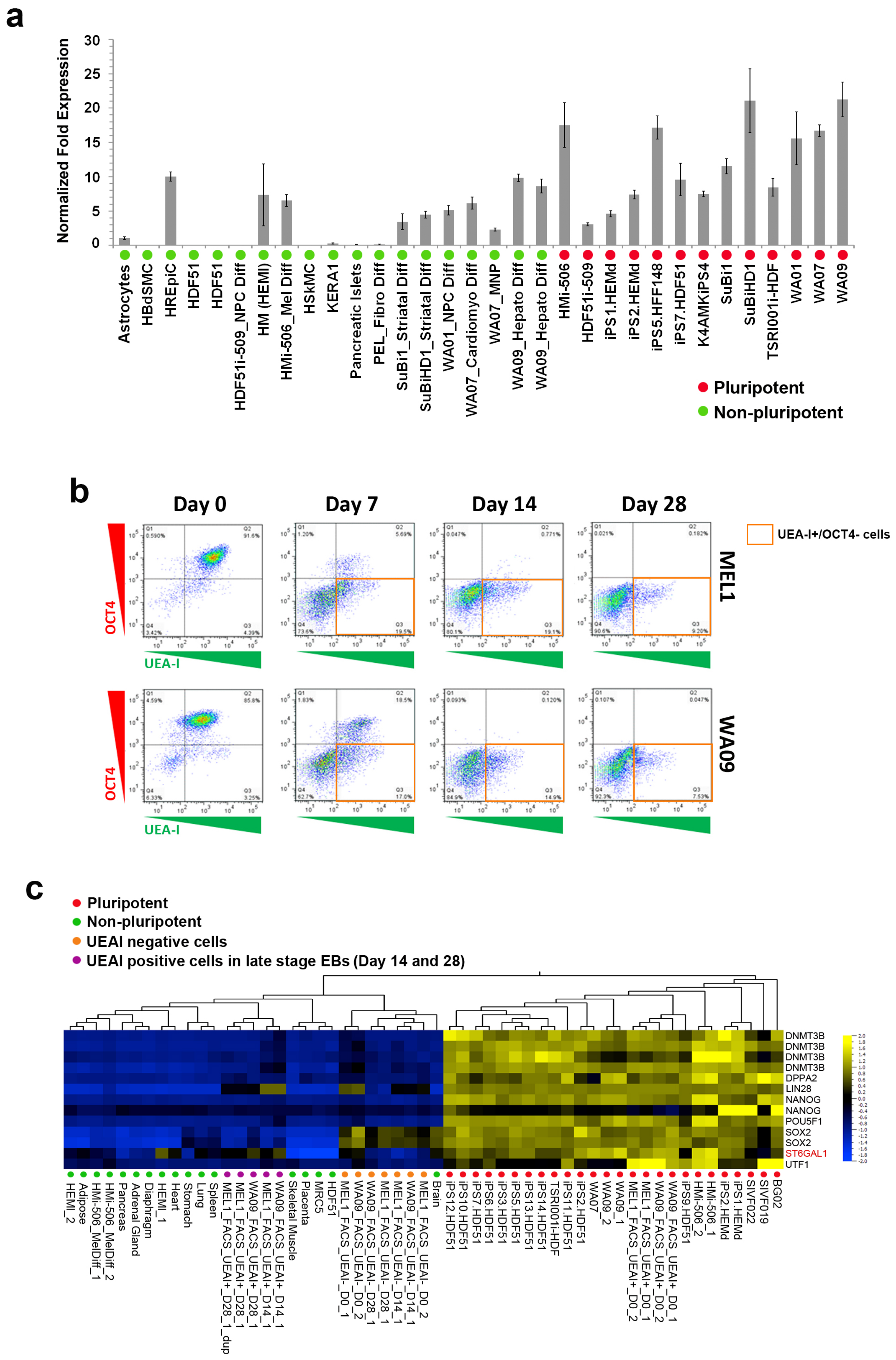

Supplementary Figure 2_Wang et. al.

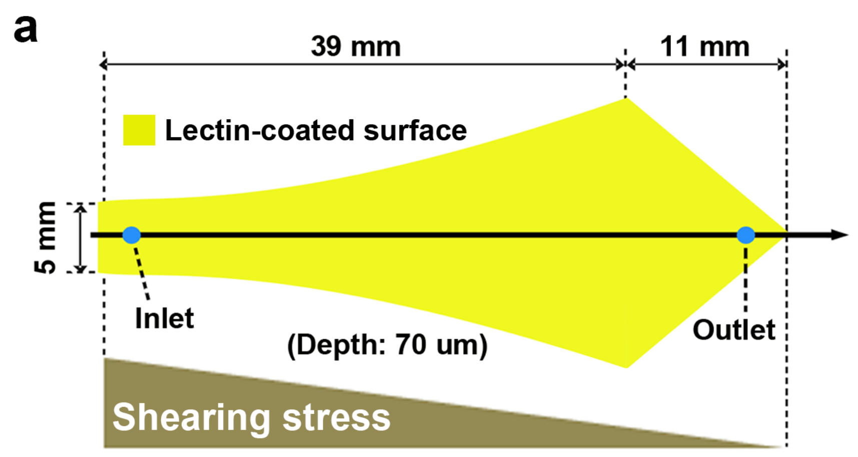

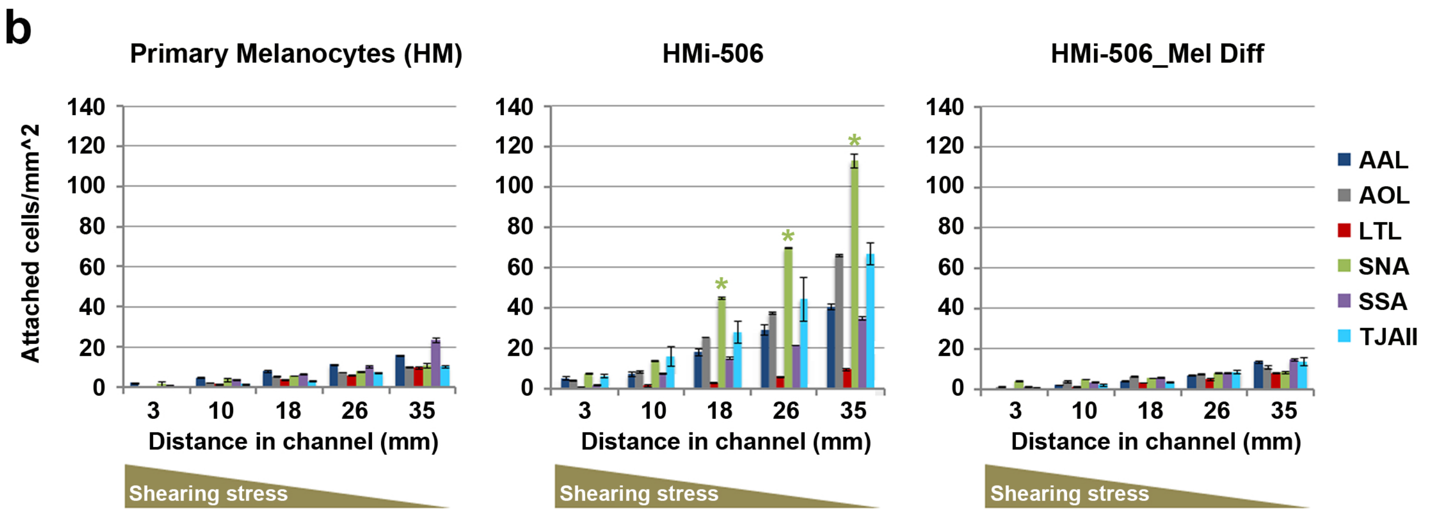

Supplementary Figure 3_Wang et. al.

a

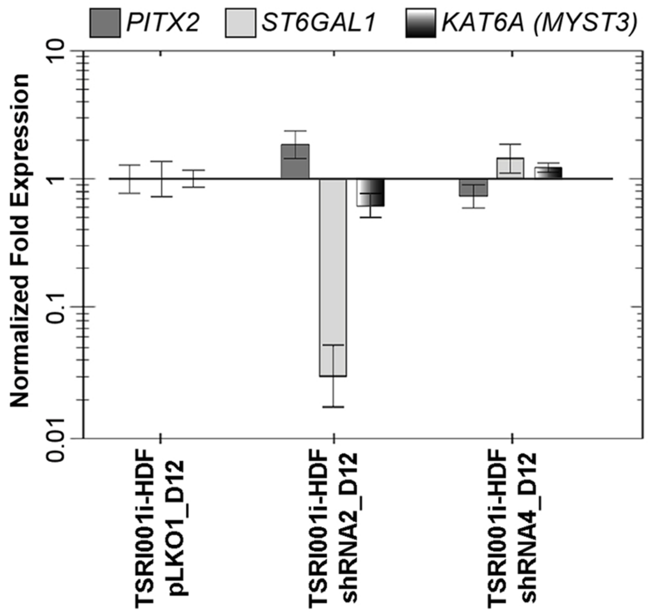

b

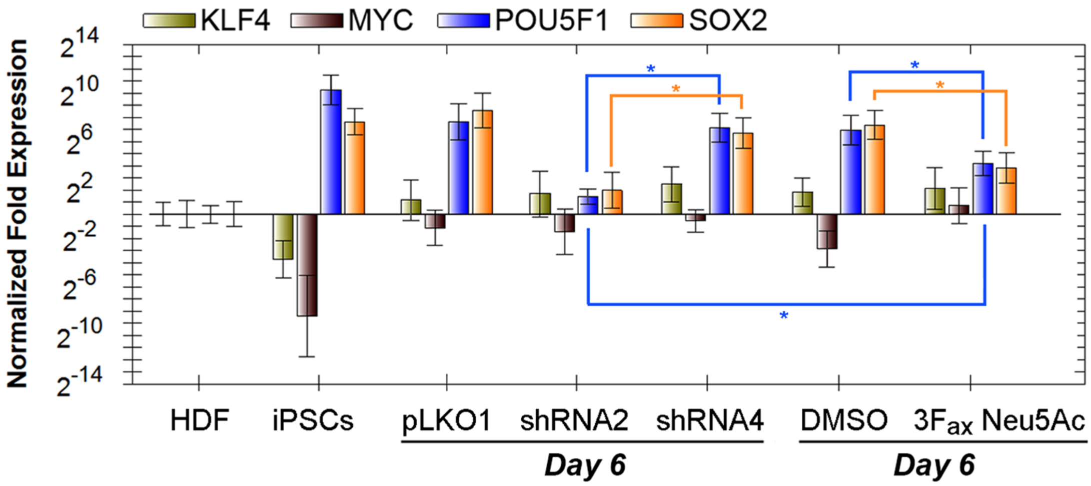

Supplementary Figure 4_Wang et. al.

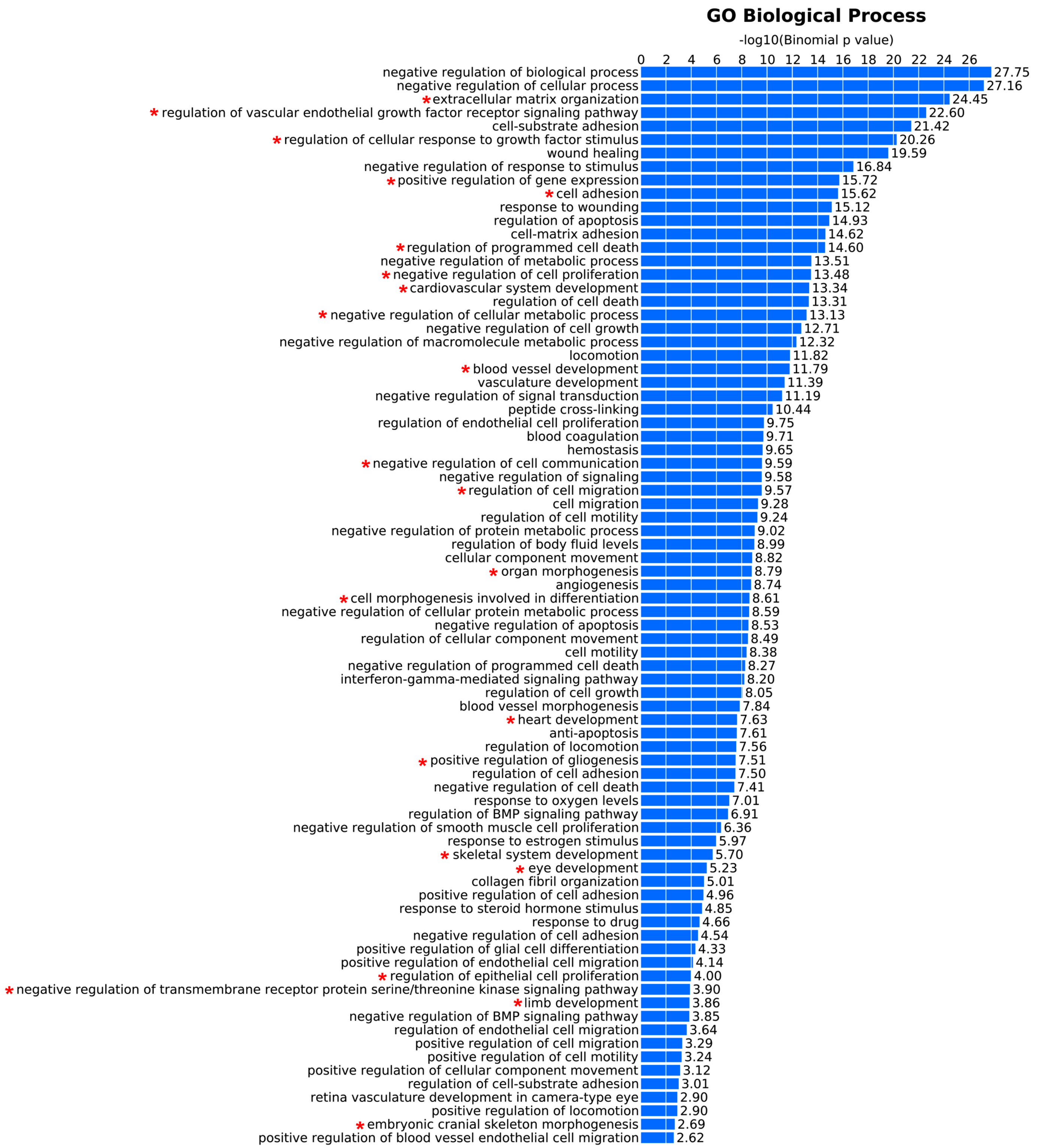

Supplementary Figure 5_Wang et. al.

a

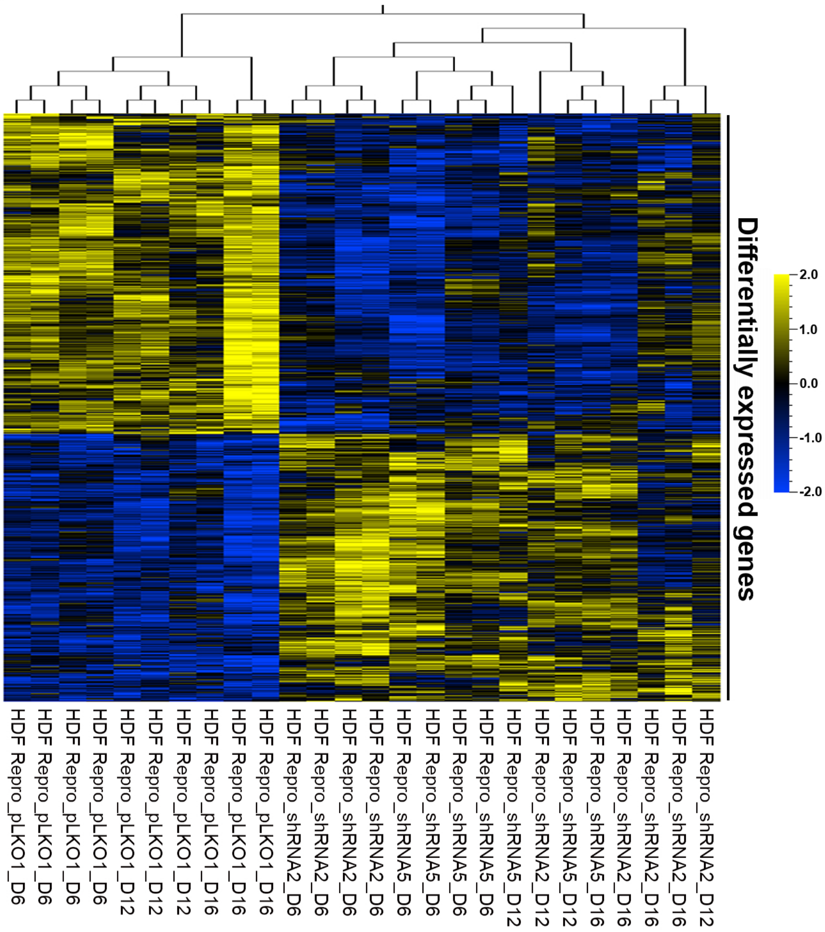

b

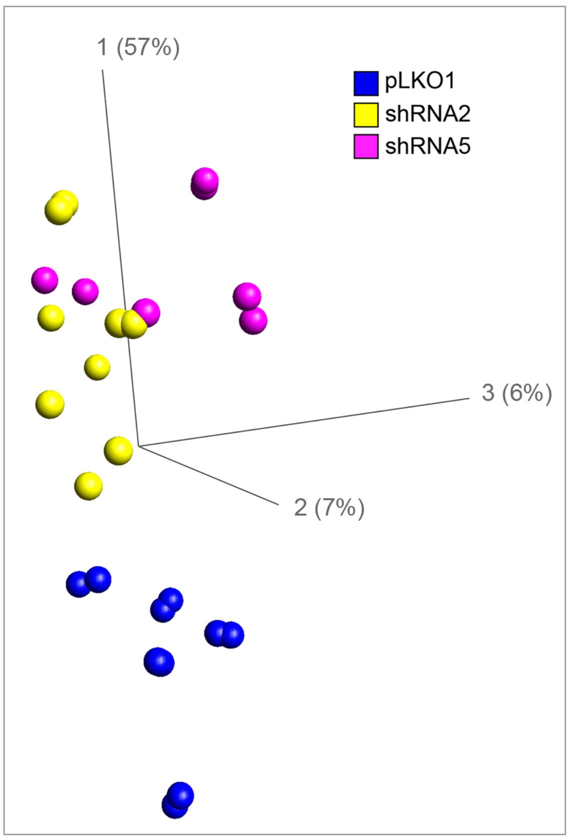

c

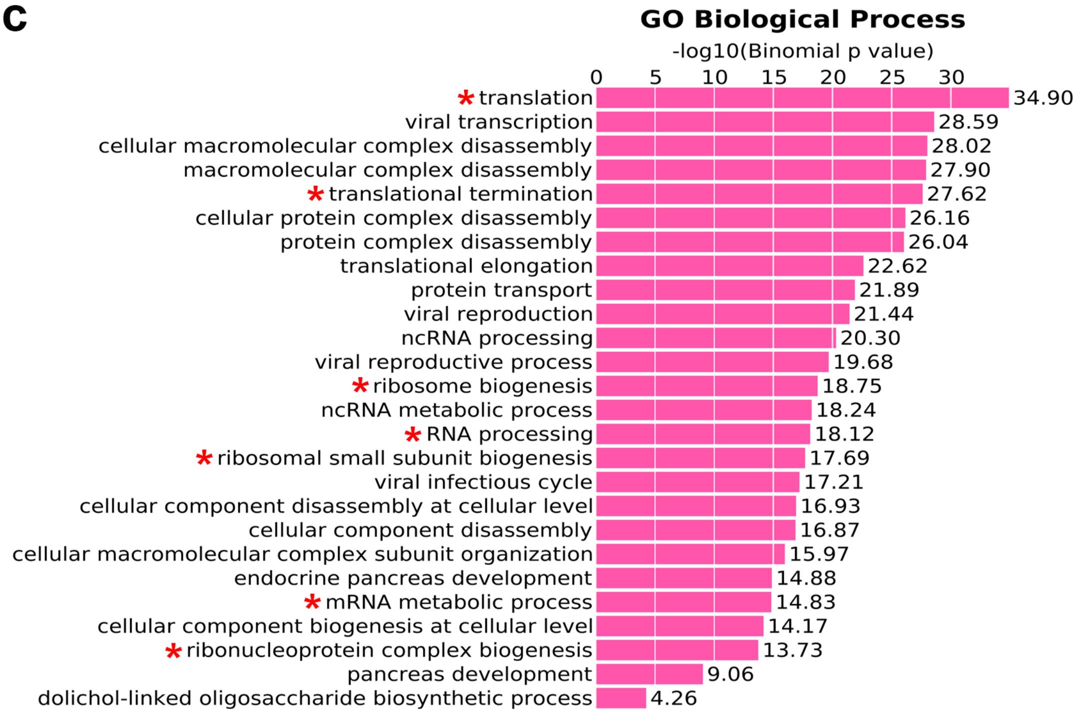

Supplementary Table 1. The list of cultured cells used in the study		
**Sample Name**	**Registry Name[1]**	**Note[2]**
**Human embryonic stem cells**		
WA07	WA07	Feeder cell-free culture on Geltrex, accutase passage[3]
WA09	WA09	Feeder cell-free culture on Geltrex, accutase passage[3]; Culture on inactivated feeder cells, collagenase passage[4]
MEL1	MEL-1	Culture on inactivated feeder cells, collagenase passage[4]
**Induced pluripotent stem cells from Human Dermal Fibroblasts (HDF)**		
TSRI001i-HDF	TSRI001i-HDF	Retrovirus-mediated reprogramming in HDF51 cells; Feeder cell-free culture on Geltrex, accutase passage [3]
HDF68i-505	TSRI68i-HDF505	Sendai virus-mediated reprogramming in HDF68 cells; Feeder cell-free culture on Geltrex, accutase passage [3]
**Induced pluripotent stem cells from Human Epidermal Melanocytes (HEM)**		
HMi-506	TSRIi-HEMl506	Sendai virus-mediated reprogramming in HM cells; Feeder cell-free culture on Geltrex, accutase passage [3]
**Cells used for reprogramming**		
HDF51 (HDF-f[5])	NA	Human dermal fibroblasts, fetal skin; Sciencell[3]
HDF68	NA	Human dermal fibroblasts, skin biopsy of adult shoulder at the Scripps Research Institute passage[3]
HM (HEMl[5])	NA	Human epidermal melanocytes, neonatal skin; Sciencell[3]

1. Name of cell line submitted to International Stem Cell Registry (U. Mass)
2. Somatic cell type, reprogramming method, culture condition, source of cells.
3. Cells cultured in Loring laboratory
4. Cells cultured in Laslett laboratory
5. Nomenclature used by the manufacturer (ScienCell Research Laboratories)

Supplementary Table 2 Genes that appeared as potential regulators for cellular pluripotency in a reported RNAi screening are differentially expressed in hPSCs with ST6GAL1 knockdown

Gene Name	Fold Change (knockdown/control)	*P* value (*F*-test)	z-score in screening[a,b]
PTPRM	1.444159	0.004339	3.222905
GNG11	2.06176	0.003924	2.284858
RPL21	1.158191	0.004043	2.414657
ALDH1L2	1.445762	0.004113	-2.42197
ATOH8	1.708084	0.002713	-2.31071
GSPT1	0.830309	0.002019	-2.31286
SLC2A12	0.706077	0.001171	-2.37161

[a] $z = (X - \mu)$/s.d., μ: mean of the negative controls (cells with non-tageting siRNA), s.d.: the standard deviation of the whole population, X: sample value calculated based on the integrated fluorescent intensity/ number of cells. Data reported in the previous publication (Ref. 26) by another group.

[b] Positive z-values indicate that fluorescence intensity in the H1 *POU5F1–GFP* reporter hESCs was increased by siRNA targeting the listed genes in the reported screening, while negative z -values indicate that fluorescence intensity in the H1 *POU5F1–GFP* reporter hESCs was reduced by siRNA targeting the listed genes.
